# Poly(methyl methacrylate) as Healing Agent for Carbon Fibre Reinforced Epoxy Composites

**DOI:** 10.3390/polym15051114

**Published:** 2023-02-23

**Authors:** Mónica Peñas-Caballero, Enrico Chemello, Antonio Mattia Grande, Marianella Hernández Santana, Raquel Verdejo, Miguel A. Lopez-Manchado

**Affiliations:** 1 Institute of Polymer Science and Technology (ICTP), CSIC, C/Juan de la Cierva, 3, 28006 Madrid, Spain; 2 Department of Aerospace Science and Technology, Politecnico di Milano, Via La Masa 34, 20156 Milan, Italy

**Keywords:** thermoplastic, coating, self-healing, carbon fibre, fracture toughness

## Abstract

Self-healing materials offer a potential solution to the problem of damage to fibre-reinforced plastics (FRPs) by allowing for the in-service repair of composite materials at a lower cost, in less time, and with improved mechanical properties compared to traditional repair methods. This study investigates for the first time the use of poly(methyl methacrylate) (PMMA) as a self-healing agent in FRPs and evaluates its effectiveness both when blended with the matrix and when applied as a coating to carbon fibres. The self-healing properties of the material are evaluated using double cantilever beam (DCB) tests for up to three healing cycles. The blending strategy does not impart a healing capacity to the FRP due to its discrete and confined morphology; meanwhile, coating the fibres with the PMMA results in healing efficiencies of up to 53% in terms of fracture toughness recovery. This efficiency remains constant, with a slight decrease over three subsequent healing cycles. It has been demonstrated that spray coating is a simple and scalable method of incorporating a thermoplastic agent into an FRP. This study also compares the healing efficiency of specimens with and without a transesterification catalyst and finds that the catalyst does not increase the healing efficiency, but it does improve the interlaminar properties of the material.

## 1. Introduction

Fibre-reinforced plastics (FRPs) are widely used in structural applications due to their exceptional mechanical properties, including high fatigue and corrosion resistance, good dimensional stability, and an excellent strength-to-weight ratio. However, despite their many advantages, FRPs are susceptible to damage from environmental, mechanical, and chemical factors, which can increase maintenance costs and require repair or replacement [1]. Self-healing materials offer a potential solution to this problem by enabling the in-service repair of composites at a lower cost, in less time, and with improved mechanical properties compared to traditional repair methods. Various strategies for self-healing materials have been developed in recent years, including the use of capsules, vascular systems, reversible covalent bonds, supramolecular interactions, and polymer blends [2]. The choice of a particular approach depends on the type of application, the stimulus required for repair, and the nature of the materials.

Capsule- and vascular-based self-healing systems are extrinsic mechanisms that involve the incorporation of self-healing agents into microcapsules or channels. When damage occurs, the capsules or channels rupture, releasing the repair agent, which infuses the fracture surfaces. The liquid healing agent is then transformed into a solid, allowing the damage to be repaired and the structural properties of the material to be restored [3]. Intrinsic self-healing systems, on the other hand, rely on the inherent properties of the polymeric material to effect the repair through reversible bonding [4]. These materials are often referred to as “mendable” polymers because they can heal damage when the material is exposed to an external stimulus, such as heat [2]. Some common reversible bonds include sulphur bonding [5], Diels–Alder chemistry [6,7], and supramolecular interactions, for example hydrogen bonding [8].

Another option is to incorporate thermoplastics into the thermoset (e.g., epoxy) matrix as polymer blends. When the thermoplastics melt under the influence of temperature, they flow into cracks, repairing them. There are two types of blends: miscible and immiscible. In the case of miscible blends, when the thermoplastic polymer dissolves in the matrix, the repair process occurs by diffusion of the polymer chains into the crack. An example is polycaprolactone (PCL) in epoxy resins cured with amine-type hardeners [9,10,11]. In the case of immiscible polymers, Cohades et al. [12] described a repair mechanism consisting of the following steps: (i) melting and volume expansion of the thermoplastic; (ii) flow of the molten thermoplastic into the damaged area; (iii) physical or chemical self-repair at the molecular level. One of the most common thermoplastics is poly(ethylene-co-methacrylic acid) (EMAA). The use of EMAA has shown high healing efficiency due to its high coefficient of thermal expansion and covalent cross-linking with epoxy resin [13]. EMAA has been incorporated into composites as discrete particles [14,15], films [16], meshes [17], filaments [18,19], or porous membranes [20]. Other thermoplastics that have been investigated for use in self-healing materials include ethylene vinyl acetate (EVA), poly(ethylene-co-glycidyl)-methacrylate (PEGMA), poly(vinyl butyral) (PVB), styrene-ethylene-butadiene copolymer (SEBS), and acrylonitrile-butadiene-styrene (ABS) [21]. Recent studies have shown that the intercalation of polymer blends between fibre layers can be used as an efficient method for self-repair. For example, Chen et al. [22] demonstrated the use of thermoplastic polyamide nanofibres as a repair agent in CFRPs. In this study, we investigated the use of PMMA as a self-healing agent in carbon fibre composites. We hypothesised a healing mechanism based on both transesterification reactions and flow-induced repair and analysed it by blending the healing agent with the matrix and as a coating on carbon fibres. Self-healing was evaluated by performing double cantilever beam (DCB) tests and examining the efficiency of the healing process over three healing cycles. We showed that the use of a PMMA coating is an effective self-healing strategy with a diffusion-based healing mechanism.

## 2. Materials and Methods

### 2.1. Materials

The epoxy resin used in this study was Resoltech 1050/1053s from Resoltech (RESOLTECH, Monaco, France), consisting of diglycidyl ether of bisphenol F (DGEBF 50–80%), diglycidyl ether of bisphenol A (DGEBA, 10–40%) and 1,6 hexanediol diglycidyl ether, and an amine-type hardener. The resin and the hardener must be thoroughly mixed at a specified ratio of 100:35 in parts by weight. This system has very low viscosity and is particularly suitable for infusion processes. The curing reaction was performed at 80 °C for 3 h according to the supplier’s specifications. Unidirectional carbon fibre tape of 12 k, density of 340 g/m^2^, and thickness of 45 μm was supplied by INP96 (INP96, S.L., Madrid, Spain). Poly(methyl methacrylate) (PMMA) from Altuglas^TM^ (TRINSEO LLC., Berwyn, PA, USA) was used as the thermoplastic healing agent. Zinc acetate (ZnAc_2_) from Sigma Aldrich (Merck, Madrid, Spain) was used as the catalyst.

### 2.2. Composite Fabrication

The addition of PMMA as healing agent was performed as follows:

1. Polymer blends: PMMA in pellet form was previously ground using a cryomill to 50- to 200-µm particles and then mixed with the resin for 15 min at room temperature with constant stirring;

2. Fibre coating: PMMA was dissolved in acetone at a concentration of 0.015 g/mL and kept in an ultrasonic bath at 50 °C for 20 min. The solution was sprayed on both sides of the fibre tape using an air spray gun. To avoid the formation of aggregates, the solution was sonicated before use. Finally, the coated carbon fibres were dried in an oven at 56 °C to remove the solvent. 

**Figure 1:**
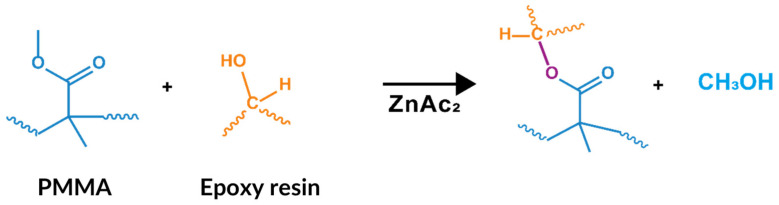
Transesterification reaction between PMMA and epoxy resin.

Healing agent concentrations of 10 and 20 wt.% were set in relation to the amount of resin in the composite material. These concentrations were chosen because they are the most commonly employed in studies using thermoplastics as healing agents in CFRPs [14,15]. The composites with plain carbon fibre or PMMA-coated carbon fibres were prepared using vacuum-assisted resin infusion moulding (VARI) (Table 1). The carbon fibres were placed unidirectionally, and the infusion was performed in the fibre direction. Four-ply laminates of 1.2–1.4 mm in thickness were prepared for flexural and interlaminar shear strength (ILSS) tests, and 14-ply laminates of 4.5–4.6 in mm thickness were used for static interlaminar fracture toughness tests. For the latter, a 13-µm Teflon film was placed along the mid-plane to initiate the delamination of the composite. All of the plies of the 4-ply laminates contained PMMA-coated fibres, whereas for the 14-ply laminates, the coated fibres were only placed in the 6 central plies. The laminates were cured at 80 °C for 3 h and then post-cured at 150 °C for 2 h in a hydraulic press to promote sheet compaction. In both methods, a catalyst (ZnAc_2_) was added to promote transesterification reactions (Figure 1) [23]. The rate of the transesterification reactions is slow and can take up to 15 h to complete. The use of metal salts, such as zinc acetate or zinc acetylacetonate, can reduce the reaction time to 2 h [23,24]. The catalyst could not be mixed directly into the resin because it precipitated and hindered the infusion process. Thus, it was incorporated onto the surface of the fibres prior to the infusion process by spraying a solution of ZnAc_2_ in ethanol (0.025 g/mL) with up to 9 wt.% with respect to the PMMA. All samples for mechanical testing were cut on a Neurtek Brillant 220 precision cutting machine.

### 2.3. Characterisation

The viscosity of the resin and its blends was evaluated in a Brookfield HA Ametek viscometer model DVNext (Brookfield, New York, USA) at 23 °C according to UNE-EN ISO 2555:2018.

The morphologies of the polymer blends, the fibre coating, and the cryogenic fracture surface of the composites were observed using an environmental scanning electron microscope (ESEM, Phillips, model XL30) (Phillips, Eindhoven, The Netherlands) with a tungsten filament and an acceleration voltage of 25 kV. Prior to observation, the samples were sputter-coated with gold/palladium (SC7640, Polaron). 

Differential scanning calorimetry with DSC 214 Polyma (Netzsch, Selb, Germany) was performed at a scan rate of 10 °C/min in the temperature range of 40 to 250 °C, following a heating/cooling/heating cycle in a nitrogen atmosphere.

The flexural properties and interlaminar shear strength (ILSS) of the uncoated and coated carbon fibre composites in the longitudinal fibre direction were measured using a 3-point bending test according to ASTM D790-03 and ASTM D2344, respectively. The specimen dimensions were 50.8 mm long and 12.5 mm wide for flexure tests and 15 mm long and 7.5 mm wide for ILSS tests. An Instron 2204 (Instron, Norwood, MA, USA) machine was used for both tests, with a load cell of 1 kN for flexural tests and 50 kN for ILSS tests, at a head speed of 1 mm/min. A minimum of 5 laminates were tested for each composite type.

The interlaminar fracture toughness of the composites was evaluated under Mode I and II static loading. For Mode I, DCB tests were performed according to ASTM D55S8 specifications. The specimen dimensions were 100 mm long and 20 mm wide, with a 40 mm long pre-crack along the mid-plane. Two metal hinges were bonded to the end of the specimen using a 2-part epoxy adhesive, Araldite 2014-2 (Ceys, Madrid, Spain) to withstand the peel force of the load cell. The test was performed by applying a tensile load to the pre-cracked end of the sample at a constant crosshead displacement of 1 mm/min using an MTS 858 mini Bionix (MTS System, Eden Prairie, MN, USA). A camera, Canon EOS 70D (Canon, Madrid, Spain) was used to follow the crack growth on the side surface of the specimens, taking pictures every 5 s. To observe the crack growth, 1 side surface of the specimens was coated with a spray developer (SKD-S2), and marks were made on the crack surface every 5 mm. Mode I interlaminar fracture toughness (*G_I_*) was calculated using the modified beam theory (MBT) method:

(1) $$G_{I}=\frac{3P\delta }{2b\;\left(a+\left|\Delta \right|\right)}\;$$

where *P* is the force; *δ* is the displacement; *a* is the crack length; *b* is the specimen width; and Δ is the effective extent of delamination, used to correct for rotation of the arms of the DCB. 

For Mode II, end notched flexure (ENF) tests were performed in accordance to ASTM D7905/D7905M. The specimen dimensions were 160 × 19 mm with a pre-crack of 45 mm. The test was performed with displacement control using an MTS 858 mini Bionix, at a crosshead rate of 1 mm/min. *G_II_* was calculated using the compliance calibration:

(2) $$G_{II}=\frac{3mP^{2}a^{2}}{2b\;}\;\;\;$$

where *P* is the force; *a* is the crack length; and *b* is the sample width.

Healing efficiency was assessed by DCB testing, as this is the most commonly used test to evaluate self-healing [25]. The delaminated composites were subjected to a healing protocol at 150 °C for 120 min, with minimal pressure applied to ensure contact between the cracked surfaces. The healing efficiency was improved by applying pressure to the delaminated composites, as it minimised the gap between the crack surfaces to be repaired [26]. The healed samples were then cooled to room temperature and re-tested under the same conditions. Self-healing efficiency was calculated as the ratio of the fracture toughness in the repaired state to that in the virgin state:

(3) $$n\;\left(\%\right)=\frac{G_{I}^{repaired}}{G_{I}^{virgin}}\;\cdot 100\;\;\;$$

The damaged surface was evaluated by microscopy to assess the interphase healing using a SEM TM3000 Tabletop Microscope (Hitachi, Tokyo, Japan) without sputter coating with gold.

## 3. Results and Discussion

**Figure 2:**
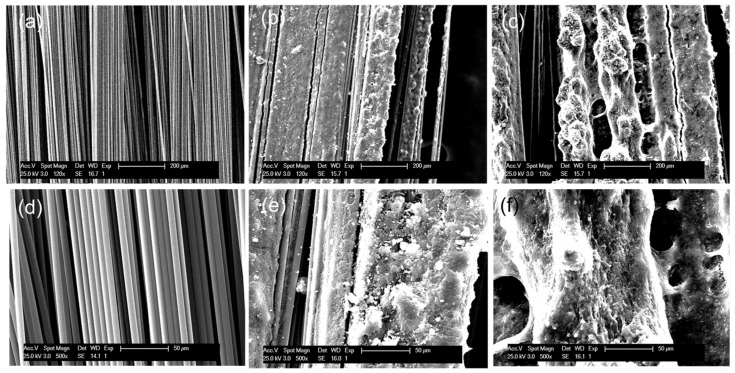
SEM images of uncoated carbon fibres (**a**,**d**) and PMAA-coated carbon fibres at 10 wt.% (**b**,**e**) and 20 wt.% (**c**,**f**).

Surface modification of fibres by spray coating is a simple and easily scalable method of introducing thermoplastic healing agents into composites, which can act as a functional sizing method. The thermoplastic was deposited onto the carbon fibre surface by spraying a solution of PMMA in acetone at room temperature. After spraying the solution, the acetone evaporated, and the PMMA precipitated onto the carbon fibres. The coating was found to be robust, and it strongly adhered to the fibre surface, allowing the fibres to be handled without loss of the thermoplastic and stored until use. SEM inspection revealed a homogeneous and uniform coating over the entire carbon fibre, increasing the roughness of the fibres (Figure 2).

**Figure 3:**
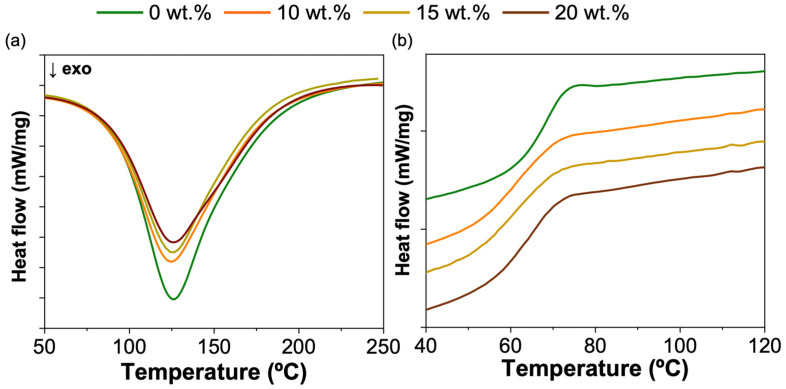
Effect of the thermoplastic agent on (**a**) cure reaction and (**b**) *T_g_* of epoxy.

Blending PMMA into the resin barely changed the curing reaction of the epoxy resin (Figure 3a) and resulted in a single *T_g_*, 3–4 °C lower than that of the pure resin (Figure 3b). Han et al. [24] reported that, at concentrations less than 25 wt.% PMMA, the epoxy-PMMA blend was miscible and exhibited a single *T_g_*.

VARI manufacturing requires a resin, the viscosity of which is such that it can flow through the distribution medium and impregnate the fibres. As expected, blending PMMA into the resin increased its viscosity, as the PMMA content in the mixture increased. However, even at a PMMA content of 20 wt.%, the viscosity of the resin was still suitable for use in the VARI process. Table 1 shows that the incorporation of PMMA resulted in a slight decrease in the carbon fibre weight fraction and an increase in the thickness of the composites. This effect was more pronounced when PMMA was added by deposition on the fibre surface. This is due to the volume occupied by the PMMA on the fibre, which increased the thickness of the laminate during vacuum densification, creating epoxy-rich interlaminar regions. Similar results have been reported in the literature when incorporating thermoplastic additives into composites and have been ascribed to the obstructed nesting of the reinforcement layers during compaction [16,26,27,28]. However, the carbon fibre content is relatively high in all laminates, with a weight fraction close to 70%.

Table 2 summarises the effect of the addition of a thermoplastic additive on the flexural and ILSS properties of FRP. In general, PMMA had a negative effect on the flexural properties of the laminates, with a greater effect on properties in the longitudinal direction. Meanwhile, the interlaminar shear strength was not as affected by the presence of the PMMA as the flexural values. It should be noted that laminates with PMMA deposited on the surfaces of the fibres showed the best properties, with only slight reductions in strength and flexural modulus. Similar reductions in mechanical performance have been reported for FRPs with the addition of thermoplastic healing agents in various configurations and have been attributed to the presence of microstructural defects in the composite, including in-plane fibre waviness, out-of-plane fibre crimping, and resin-rich regions [14,29,30,31]

The interlaminar properties were studied using the DCB and ENF test methods. Similar to the previous mechanical results, the Mode I and Mode II interlaminar fracture toughness values decreased as the PMMA concentration increased (Table 3). The results showed that the coating had a greater effect than the blend, which could be attributed to a reduction in adhesion between the carbon fibres and the epoxy resin as a result of the PMMA coating and to the fact that the PMMA and epoxy resin formed an immiscible phase, which may result in weaker interactions between the two polymers. Previous studies have ascribed similar reductions in fracture toughness to changes in the stiffness of the epoxy due to the incorporation of the thermoplastic phase and to the intrinsic toughness behaviours of the blends in which the thermoplastic is confined within the epoxy resin [30].

**Figure 4:**
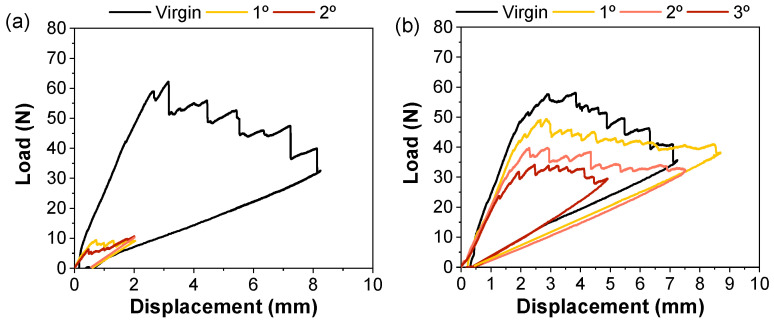
Load–displacement curves of the DCB test for up to 3 healing cycles of (**a**) 10 wt.% and (**b**) 20 wt.% PMMA-coated samples.

**Figure 5:**
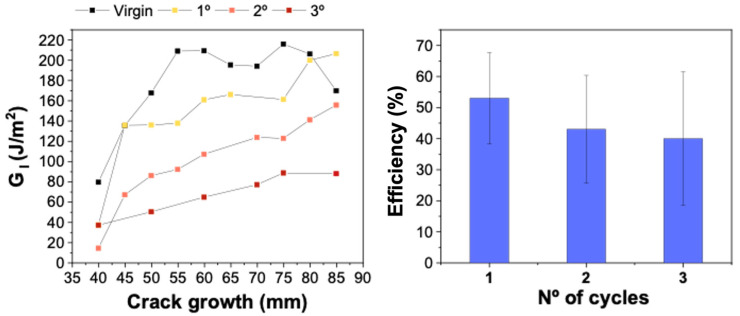
Mode II crack growth resistance curves (**left**) and efficiency for different cycles of healing for 20 wt.% PMMA-coated samples (**right**).

The healing efficiency of the PMMA-coated composite was examined using Mode I fracture toughness. Blending PMMA into the epoxy resulted in no healing capacity, which was attributed to the morphology of the thermoplastic phase, which hindered its expansion [28]. Meanwhile, the PMMA-coated samples demonstrated healing capacity. Figure 4 shows the load versus displacement curves for the DCB test after different repair cycles. It can be observed that the crack energy release rate was lower in the healed samples, indicating that the crack propagated at lower load levels. The healing efficiency of C10 was found to be less than 15% and increased significantly for the C20 sample, in which the load after healing was comparable to the virgin sample. Figure 5 shows the *G_I_* and healing efficiency for different healing cycles of the C20 sample. After the first cycle, efficiency of 53% was achieved for interlaminar fracture toughness, which is a significant result for PMMA as a healing agent. The efficiency of the second cycle was 43%, and the third cycle showed a slight decrease to 40%. Although slight decreases in flexural properties and interlaminar fracture toughness were observed for the 20 wt.% PMMA sample compared to the uncoated samples, the increase in healing efficiency was significant compared to the samples with 10 wt.%, which is a remarkable result even after three healing cycles. These efficiencies were lower than those reported for other thermoplastics, such as EMAA [32,33]. However, these findings open up possibilities of further investigating the potential of PMMA to enhance the healing capabilities of composites.

**Figure 6:**
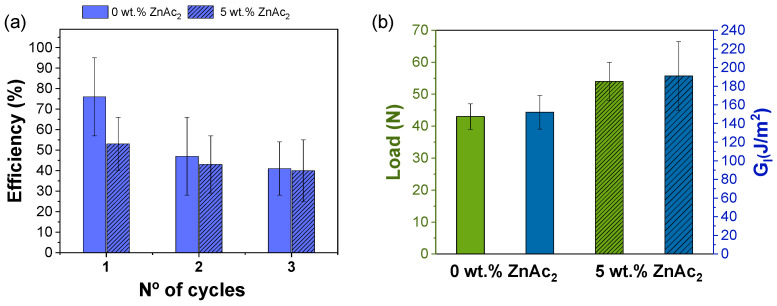
(**a**) Comparison of the efficiencies obtained for *G_I_* of composites without and with catalyst. (**b**) Maximum load and *G_I_* values for composites without and with catalyst.

The healing mechanism was initially thought to involve both flow and transesterification reactions, in which the alcohol group of the epoxy resin reacted with the ester group of PMMA at high temperatures (150 °C) [24]. To evaluate the role of the transesterification reaction in the healing process, laminates were prepared without the ZnAc_2_ catalyst. The results, shown in Figure 6, indicated that the healing efficiency increased to 76% for *G_I_* when the catalyst was removed. The second and third cycles showed similar results for the samples with and without the catalyst. Therefore, these results suggested that the transesterification reaction was not the primary mechanism driving the healing process. However, when comparing the mechanical performance of the samples (Figure 6b), it was observed that the maximum load decreased from 52 N to 42 N and the interlaminar fracture resistance decreased from 191 J/m^2^ to 149 J/m^2^ in the samples without the catalyst. This outcome suggests that the catalyst increased the adhesion at the interface between the epoxy resin and PMMA during the post-curing process.

**Figure 7:**
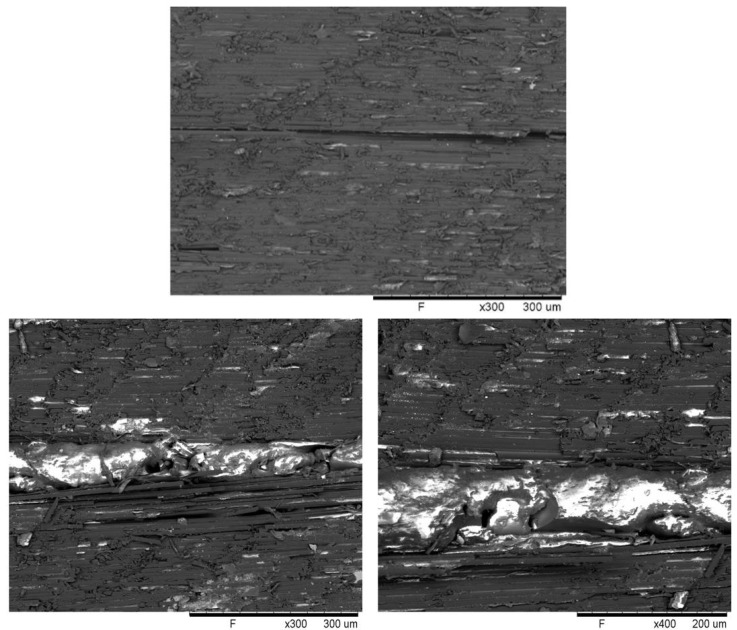
SEM images of the fracture surface without PMMA (**top**) and the repaired fracture PMMA-coated composite at different magnifications (**bottom**).

SEM examination of the fracture surface revealed that the crack was completely filled with PMMA (Figure 7). This behaviour confirmed the previously discussed mechanism underlying the healing process: when exposed to high temperatures, the thermoplastic PMMA reduced its viscosity, allowing it to flow into the volume of the crack and fill it. On cooling, the thermoplastic solidified, mechanically joining the two surfaces.

## 4. Conclusions

This study demonstrated the ability of PMMA to be used as a healing agent by incorporating it as a coating on carbon fibres. The deposition of the healing agent on the surface of the fibres is a simple and easily scalable method for the production of self-healing FRP. The PMMA formed a homogenous coating over the fibres when it was sprayed as a solution in acetone. The flexural and ILSS properties were slightly reduced in the coating system, but the interlaminar fracture toughness was significantly reduced. Despite this effect, the 20 wt.% PMMA-coated composites exhibited high healing efficiency over three cycles. This study suggests that the main mechanism of healing is the softening of PMMA during the healing cycle and that the transesterification reaction improves the interaction between PMMA and the epoxy resin, resulting in better initial interlaminar properties. Further studies should be performed to understand the effect of PMMA on fracture mechanisms and the role of catalysts in improving the initial mechanical properties.

## Figures and Tables

**Table 1:** Prepared laminates and their physical properties.

No. of Fibre Plies	Laminate *	Carbon Fibre Fraction (wt.%)	Thickness (mm)
4	B0	72	1.3 ± 0.1
	B10	70	1.4 ± 0.1
	B20	70	1.47 ± 0.04
	C0	72	1.4 ± 0.1
	C10	67	1.4 ± 0.1
	C20	64	1.57 ± 0.05
14	B0	75	4.2 ± 0.2
	B10	75	4.1 ± 0.1
	B20	74	4.2 ± 0.2
	C0	74	4.1 ± 0.1
	C10	72	4.5 ± 0.1
	C20	70	4.7 ± 0.1

* B and C stand for blend and coating, respectively. The C0 laminate was subjected to the same acetone spraying and drying protocol as the C10 and C20 laminates.

**Table 2:** Flexural and ILSS properties of composites with PMMA.

Laminate	Flexural				ILSS
	Longitudinal		Transversal		Strength, MPa
	Modulus, GPa	Strength, MPa	Modulus, GPa	Strength, MPa	
B0	71 ± 5	960 ± 80	6.0 ± 10	100 ± 20	41 ± 5
B10	46 ± 8	730 ± 90	7.4 ± 0.4	89 ± 8	53 ± 2
B20	23 ± 4	430 ± 70	7.0 ± 1.0	84 ± 7	47 ± 3
C0	66 ± 4	940 ± 60	6.3 ± 0.9	85 ± 5	46 ± 5
C10	57 ± 7	830 ± 30	5.9 ± 0.8	76 ± 7	48 ± 3
C20	54 ± 4	800 ± 80	6.2 ± 0.5	83 ± 5	52 ± 5

**Table 3:** Values of Mode I and II fracture toughness properties.

Laminate	*G_I_* (J/m^2^)	*G_II_* (J/m^2^)
B0	1140 ± 20	1135.0 ± 0.4
B10	900 ± 100	639.4 ± 0.1
B20	830 ± 90	530.4 ± 0.4
C0	850 ± 30	729.9 ± 0.7
C10	200 ± 10	296.9 ± 0.2
C20	190 ± 40	267.9 ± 0.3

## Data Availability

The data that support the findings of this study are available on request from the corresponding author, M.A. López-Manchado.
